# Diagnostic discrepancies in clinical practice: An autopsy study in patients with heart failure

**DOI:** 10.1097/MD.0000000000005978

**Published:** 2017-01-27

**Authors:** Victor Sarli Issa, Layara Fernanda Lipari Dinardi, Thiago Vicente Pereira, Lyna Kyria Rodrigues de Almeida, Thaisa Silveira Barbosa, Luiz Alberto Benvenutti, Silvia Moreira Ayub-Ferreira, Edimar Alcides Bocchi

**Affiliations:** Heart Institute (InCor) do Hospital das Clínicas da Faculdade de Medicina da Universidade de São Paulo, São Paulo (SP), Brazil.

**Keywords:** autopsy, cardiomyopathy, heart failure, necropsy

## Abstract

Autopsies are the gold standard for diagnostic accuracy; however, no recent study has analyzed autopsies in heart failure (HF).

We reviewed 1241 autopsies (January 2000–May 2005) and selected 232 patients with HF. Clinical and autopsy diagnoses were analyzed and discrepancies categorized according to their importance regarding therapy and prognosis.

Mean age was 63.3 ± 15.9 years; 154 (66.4%) patients were male. The causes of death at autopsy were end-stage HF (40.9%), acute myocardial infarction (17.2%), infection (15.9), and pulmonary embolism 36 (15.5). Diagnostic discrepancies occurred in 191 (82.3%) cases; in 56 (24.1%), discrepancies were related to major diagnoses with potential influence on survival or treatment; pulmonary embolism was the cause of death for 24 (42.9%) of these patients. In 35 (15.1%), discrepancies were related to a major diagnosis with equivocal influence on survival or treatment; in 100 (43.1%), discrepancies did not influence survival or treatment. In multivariate analysis, age (OR: 1.03, 95% CI: 1.008–1.052, *P* = 0.007) and presence of diabetes mellitus (OR: 0.359, 95% CI: 0.168–0.767, *P* = 0.008) influenced the occurrence discrepancies.

Diagnostic discrepancies with a potential impact on prognosis are frequent in HF. These findings warrant reconsideration in diagnostic and therapeutic practices with HF patients.

## 1 Introduction

Diagnostic errors are considered an important and avoidable source of morbidity and mortality.^[1]^ A missed or delayed diagnosis may impact on patient safety and also result in increased direct and downstream costs.^[2]^ Despite the recognition that a correct diagnosis is fundamental to patient care, systematic efforts to study diagnostic errors have been limited.^[3]^ Autopsies are considered “gold standard” for providing a definitive diagnosis,^[4]^ and have been used to compare in vivo and postmortem diagnoses. The results of such an analysis indicate that discrepancies remain at a 10% rate, even in face of advances in diagnostic techniques^[5]^; these findings are sustained when data are adjusted for time and geographical variations.^[6]^

Even though heart failure (HF) is a prevalent cardiovascular condition^[7]^ associated with a high mortality rate^[8]^ and a recognized source of diagnostic errors,^[9]^ no recent study has systematically analyzed the causes of death in patients who undergo autopsy. Current knowledge regarding the causes and mechanisms of death in patients with HF is mostly based on epidemiological studies and clinical trials.^[10]^ Importantly, diagnostic discrepancies between in vivo and postmortem diagnoses have not been explored in this population.

Thus, we hypothesized that the study of autopsy findings in patients with HF might contribute to the further understanding of the mechanisms and causes of death, and that the search for diagnostic discrepancies could offer an additional perspective to guide therapy for a condition associated with a high mortality rate.

## 2 Methods

### 2.1 Objectives

The main objective of our study was to describe the causes of death of patients with HF who underwent autopsy. Additionally, we sought to study the occurrence of discrepancies between in vivo and postmortem diagnoses, explore the conditions associated with a higher chance of diagnostic discrepancies, as well as the causes of death in particular clinical scenarios, namely, in patients with sudden death and in patients with preserved ejection fraction.

### 2.2 Study design

This was a retrospective study based on the analysis of autopsies performed in a university hospital dedicated to cardiology from January 2000 through May 2005. The protocol was reviewed and approved by the Institutional Ethics Committee (CAPPesq 6915, protocol 0119/11). Patient's records and autopsy reports were reviewed, and diagnoses were categorized according to previous criteria^[11,12]^ in: primary cause of death; diseases related to death; diseases unrelated to death; etiology of HF. The primary cause of death was defined as the condition that led directly to death, excluding final events of a terminal disease. Diseases related to death were defined as contributing conditions to the primary cause of death, complications of the primary cause of death, or other diseases that contributed to death. The diagnoses were reviewed by 3 investigators; one of whom was a clinical cardiologist with experience in the care of HF patients. In the event of uncertainty regarding a clinical diagnosis, a second experienced cardiologist was consulted. In the event of uncertainty regarding an autopsy diagnosis, an experienced pathologist dedicated to cardiac pathology was consulted.

The analysis of autopsy data was based on an electronic database where final autopsy reports are stored; information regarding age, sex, date of hospital admission, date of death, and autopsy diagnoses were retrieved for all patients. The analysis of clinical data was based on paper medical records, and information regarding the beginning of symptoms, previous morbid conditions, etiology of HF, and all diagnoses related to death were retrieved.

### 2.3 Definitions

The diagnosis of HF at autopsy was based exclusively on morphology and pathology findings that included organ lesions secondary to venous congestion (either systemic or in the lungs) or systemic low output state; these findings were considered along with the presence of significant cardiac disease.

Even when not explicitly registered, a clinical diagnosis was presumed when specific treatment was administered for a given condition. Sudden death was defined as unexpected out-of-hospital loss of consciousness in patients who arrived at the hospital in cardiac arrest; unexpected in-hospital death in patients who were considered hitherto clinically stable; and death consequent to cardiac arrhythmia (ventricular tachycardia, ventricular fibrillation, advanced atrioventricular block). Terminal arrhythmias in patients with end-stage HF were categorized as HF death. Death as a consequence of cardiogenic shock after acute myocardial infarction was categorized as acute myocardial infarction death.^[13]^ The cause of death was considered undetermined when no morphological explanation for death was found in the autopsy report.

At autopsy, pulmonary embolism was considered to be the cause of death of a patient if one of the following conditions were met: presence of a thrombus in the pulmonary artery or one of its main branches whose size was deemed sufficient to cause critical hemodynamic or respiratory distress; presence of pulmonary infarction whose size was deemed sufficient to cause critical hemodynamic or respiratory distress; presence of multiple thrombus throughout the pulmonary tree in an extension deemed sufficient to cause critical hemodynamic or respiratory distress; additionally, the diagnosis of pulmonary embolism was considered in the presence of clinical–pathological plausibility, and absence of another cause for the death.

### 2.4 Patients

We reviewed 1241 autopsies (January 2000/May 2005) and selected 878 patients who had in the autopsy report one of the following diagnoses: HF, cardiomyopathy, or cardiogenic shock. We excluded patients who underwent surgical or percutaneous cardiac interventions during the hospital admission in which death occurred (n = 128), patients under 18 years old or with congenital heart diseases (n = 238), patients with pericardial diseases (n = 12), and patients without retrievable clinical data (n = 268). Altogether, 232 autopsies were included in the study and were analyzed.

### 2.5 Autopsies

At our institution autopsies are performed in patients with in-hospital death, as indicated by the attending medical team, unless family consent cannot be obtained. Patients under palliative care are not systematically submitted to autopsies. At the time autopsies are performed, pathologists have access to patient charts and to a summary report of the case filled out by the attending physician, describing the clinical conditions related to the death. In all autopsies, the heart, lungs, kidneys, brain, liver, and spleen are examined; whenever macroscopic inspection suggests specific organ involvement, en bloc extraction of thoracic and/or abdominal organs is performed. Tissue samples are retrieved based on clinical data and on macroscopic inspection and include, at the least, kidneys, liver, heart, and lungs. Autopsy reports are standardized in topics according to a diagnostic hierarchy that takes into consideration both the logical sequence of events that led to death and the organs involved.

During the study period, there were 67,364 hospital admissions and 5273 in-hospital deaths (mortality rate 7.8%); 1241 (23.5%) of these patients underwent autopsy. The rate of autopsies relative to the number of deaths was 25.1% in year 2000, 23.6% in 2001, 22.5% in 2002, 25.6% in 2003, 21.1% in 2004, and 23.4% in 2005.

### 2.6 Comparison of clinical and autopsy diagnosis

Clinical and autopsy diagnoses were compared and categorized according to criteria initially proposed by Goldman et al^[14]^ and further modified.^[15,16]^ The categorization is based on the presence of discrepancies between in vivo and postmortem diagnoses, as well as in their influence over the events that led to death. A discrepancy was defined as the presence of a diagnosis during the autopsy that was not made during life.

The primary cause of death and the conditions related to death were categorized as a *major diagnosis*; conditions unrelated to death were categorized as a *minor diagnosis*. Discrepancies involving major diagnoses were further divided into classes I and II; discrepancies involving minor diagnoses were further divided into classes III and IV. The following criteria were used to define these classes: class I: discrepancy related to a major diagnosis with potential negative influence on survival and direct impact on treatment; class II: discrepancy related to a major diagnosis with equivocal influence on survival and no impact on treatment (therapy had already been administered even though the diagnosis was unknown or therapy was not available at the time); class III: discrepancy related to a minor diagnosis, that was not directly related to death, but was symptomatic and needed treatment or would have eventually affected the prognosis; class IV: discrepancy related to an occult condition of possible epidemiological or genetic interest. The absence of discrepancies was categorized as class V.

### 2.7 Statistical analysis

Categorical variables were described as absolute value and percentage; continuous variables were described as mean and standard deviation. Comparison of proportions between groups was performed with the *χ*^2^ test and comparison of means was performed with Student *t* test. Multivariate analysis was performed with logistic regression. The Hosmer–Lemershow test demonstrated that the model was adjusted after stepwise variable selection; we included in the model variables with a *P*-value in univariate analysis less than 0.1. *P* values less than 0.05 were considered significant.

## 3 Results

The analysis of 232 patients generated 733 diagnoses (3.3 diagnoses per patient); clinical characteristics of patients are depicted in Table 1. The main causes of HF were ischemic heart disease in 95 (40.9%) patients, Chagas’ cardiomyopathy in 39 (16.8%), dilated cardiomyopathy in 24 (10.3%), and arterial hypertension in 22 (9.5%). In most patients, the admission diagnosis was decompensated HF (25.4%) and cardiogenic shock (19.4%), followed by acute myocardial infarction (12.5%), sudden death (9.1%), and infections (7.3%). Atrial fibrillation had a high prevalence (60.2%). The autopsy of patients revealed that the main causes of death were HF (40.9%), acute myocardial infarction (17.2%), infection (15.9), and pulmonary embolism 36 (15.5) (Table 2).

**Table 1:**
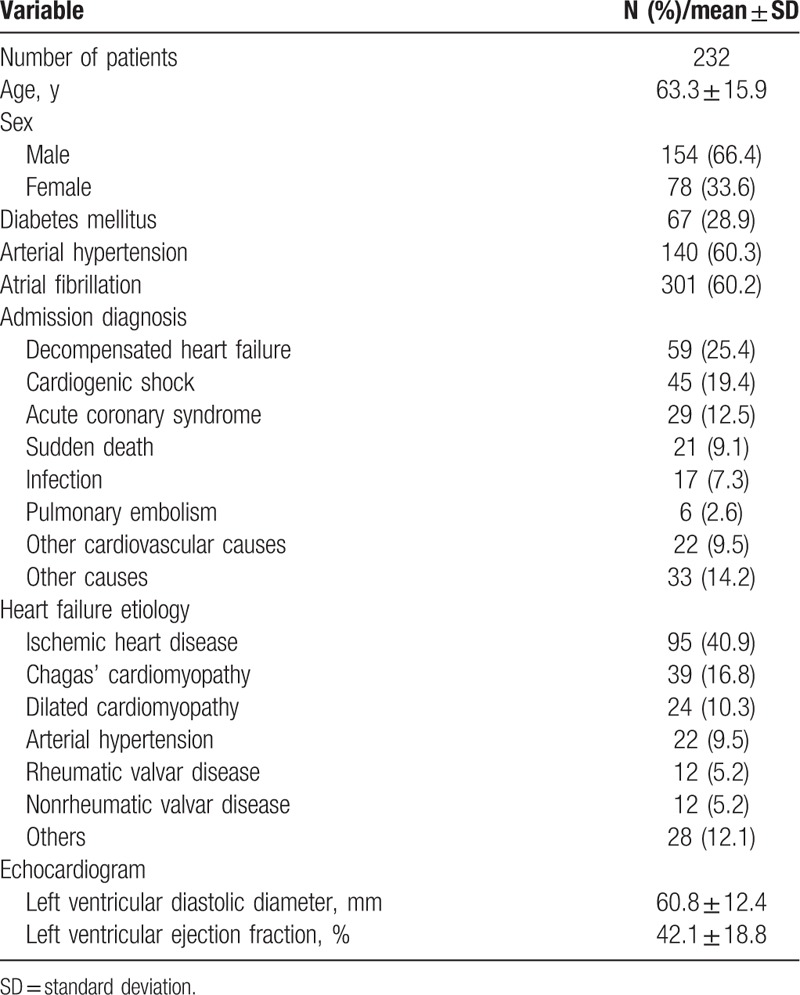
Characteristics of patients based on clinical data.

**Table 2:**
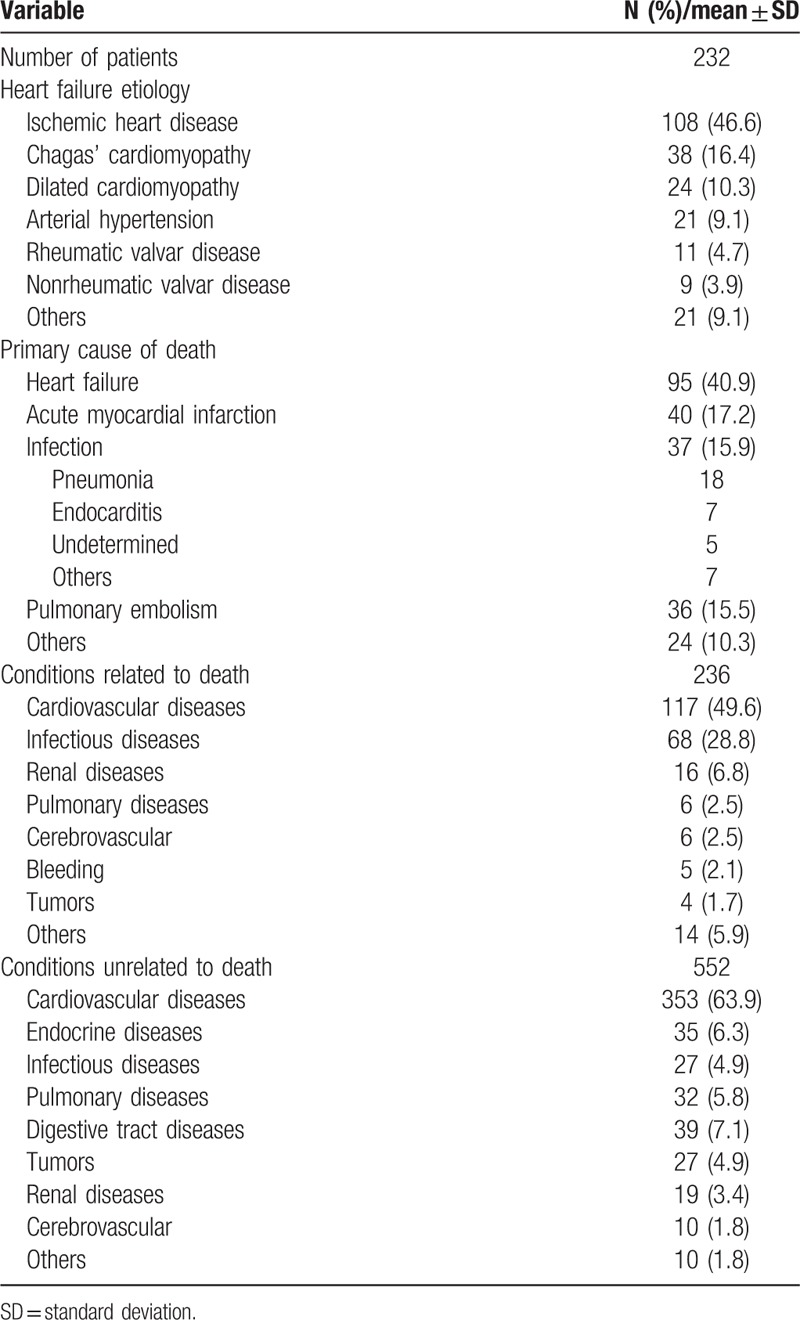
Characteristics of patients based on autopsy data.

### 3.1 Comparison of clinical and autopsy data

**Figure 1:**
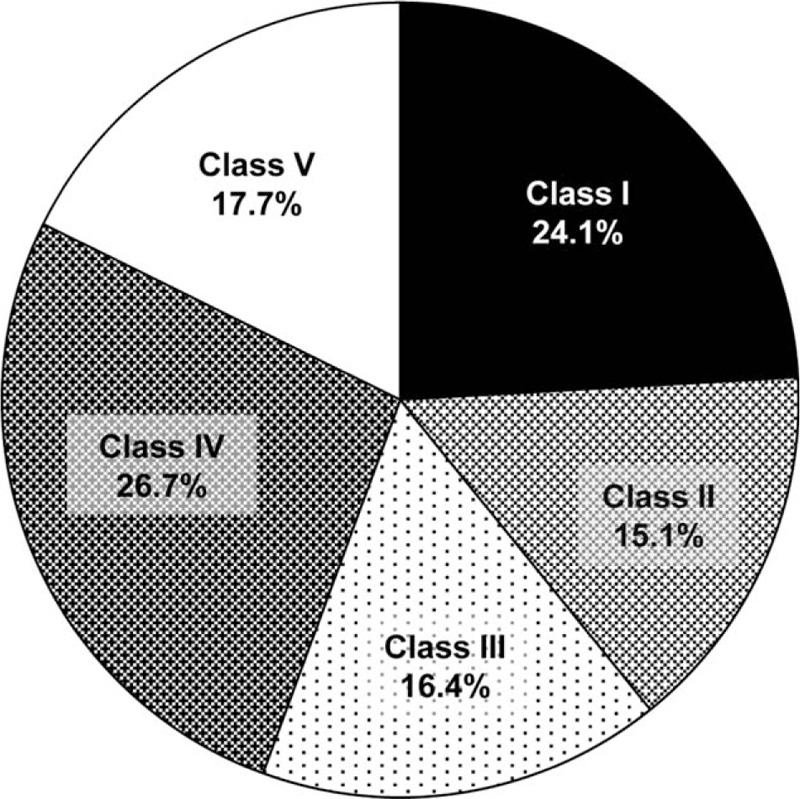
Distribution of diagnostic discrepancies.

**Figure 2:**
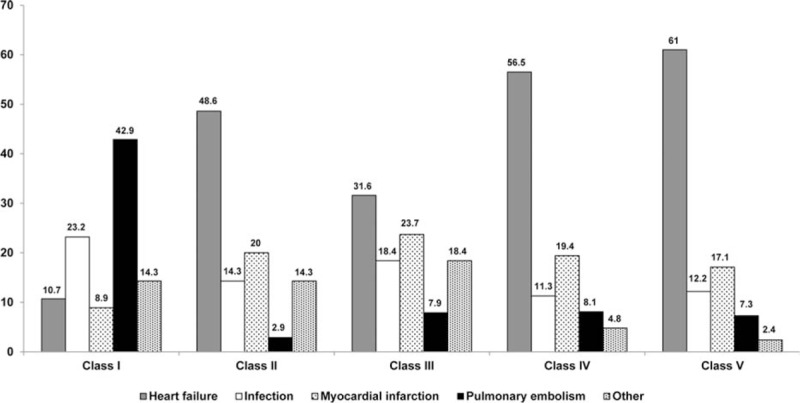
Distribution of the causes of death according to the occurrence of diagnostic discrepancies.

Discrepancies between clinical and autopsy diagnoses occurred in 191 (82.3%) patients (Fig. 1): class I discrepancies occurred in 56 (24.1%) patients, class II in 35 (15.1%), class III in 38 (16.4%), and class IV in 62 (26.7%). The distribution of discrepancies according to the cause of death is depicted in Fig. 2. Pulmonary embolism was the cause of death of 24 (42.9%) patients with class I discrepancies, and infections were the cause of death of 13 (23.2%) patients.

Compared to patients without major discrepancies (classes III, IV, and V), the patients with classes I and II discrepancies had a tendency to be older (65.4 ± 15.2 years vs 61.9 ± 16.2 years, *P* = 0.09), had a shorter length of hospital admission (6.7 ± 8.9 days vs 10.4 ± 16.5 days, *P* = 0.05), lower frequency of diabetes mellitus (20.9% versus 34%, *P* = 0.038), and a tendency toward having smaller left ventricular diastolic diameter (60.3 ± 13.4 mm vs 63.7 ± 11.1 mm, *P* = 0.06) (Table 3). When these variables were analyzed in a logistic regression model, only age and the presence of diabetes remained as statistically significant variables. The presence of diabetes decreased the risk for the occurrence of a discrepancy involving a major diagnosis (OR: 0.359, 95% CI: 0.168–0.767, *P* = 0.008); in contrast, increasing age determined higher risk for major discrepancies (OR: 1.03, 95% CI: 1.008–1.052, *P* = 0.007).

**Table 3:**
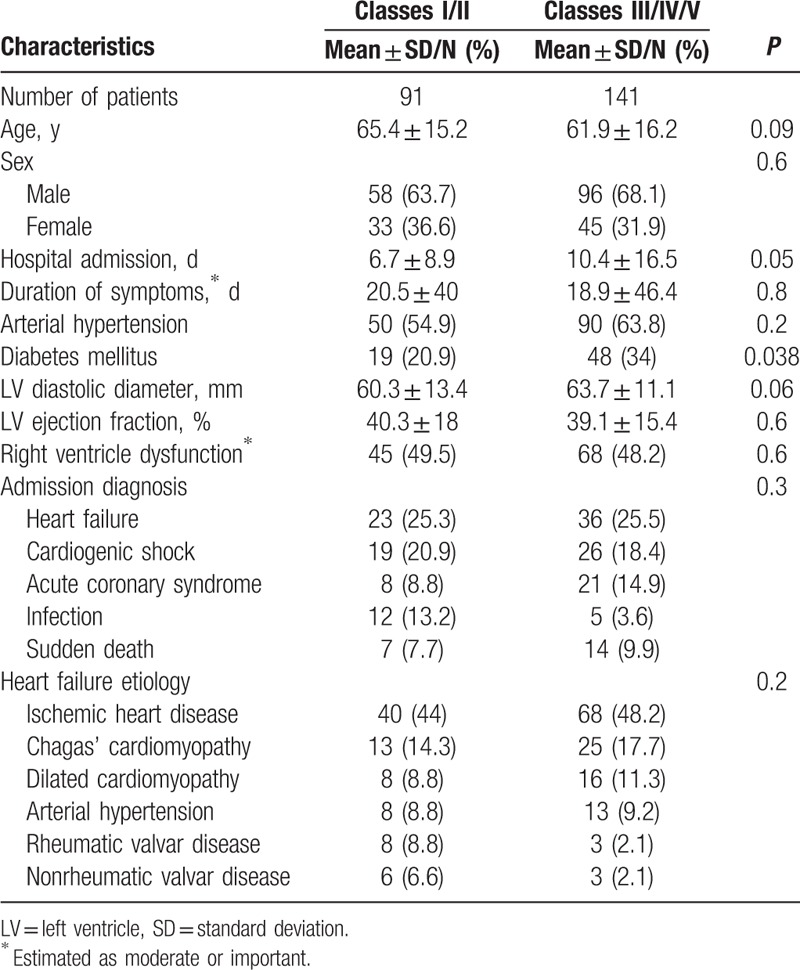
Clinical characteristics of patients according to the presence of discrepancies.

### 3.2 Analysis of patients with sudden death

Among the 232 studied patients, 32 (13.8%) had experienced sudden death; the mean age of these patients was 62.8 ± 15.1; 21 (65.6%) were men and 11 (34.4%) women; the main HF etiologies were ischemic heart disease in 19 (59.4%), Chagas’ cardiomyopathy in 7 (21.9%), arterial hypertension in 3 (9.4%), dilated cardiomyopathy in 2 (6.3%), and amyloidosis in 1 (3.1%). The autopsy identified a specific cause of death in 14 (43.7%) patients; in 13 (40.6%) patients there were only signs of HF and systemic hypoperfusion, and in 5 (15.6%) patients no morphologic findings could be associated with the cause of death (Table 4). Interestingly, in 7 of the 13 patients who had only signs of HF, an arrhythmic cause for death had been identified in life (ventricular tachycardia or ventricular fibrillation in 6 patients and atrioventricular block in 1 patient).

**Table 4:**
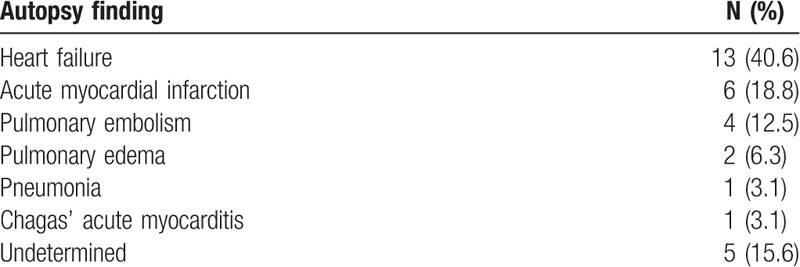
Causes of death at autopsy in patients with sudden death.

### 3.3 Analysis of patients according to left ventricle function

The left ventricular ejection fraction as measured by echocardiography could be retrieved in 202 patients; in 138 (68.3%) the ejection fraction was less than 40%. We compared clinical and autopsy findings according to ejection fraction (Table 5). As compared to patients with ejection fraction > 40%, patients with ejection fraction ≤40% were similar in age (64.7 ± 12.3 years vs 61 ± 15.3 years, respectively, *P* = 0.4), had a higher proportion of male patients (73.9% vs 26.1%, *P* = 0.009), and had a different distribution of HF etiology. The differences in the distribution of the causes of death were not statistically significant. Additionally, the proportion of patients with discrepancies involving a major diagnosis (classes I or II) was similar for both groups (39.1% and 43.7%, *P* = 0.3).

**Table 5:**
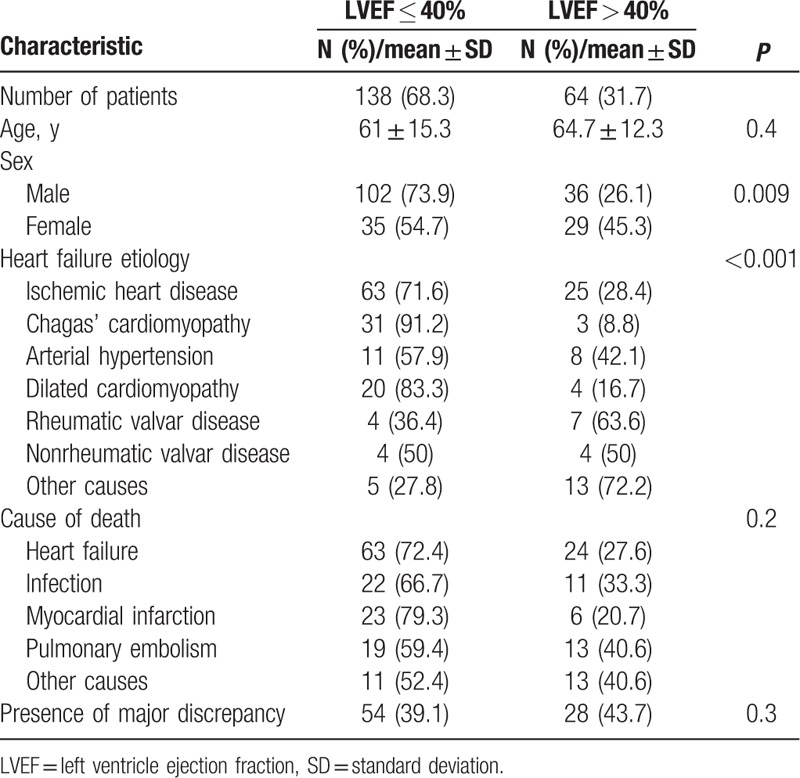
Characteristics of patients according to ejection fraction.

## 4 Discussion

To the best of our knowledge, this is the first study to report on autopsy findings in a large population of patients with HF and to compare the in vivo and postmortem diagnoses. We found that the rate of diagnostic discrepancies with a possible impact on therapy and prognosis is high in this setting, and that this occurrence is influenced by patient characteristics, namely age and the presence of diabetes mellitus; pulmonary embolism was the main cause of death among patients with discrepancies involving the major diagnosis. Additionally, our results show that progressive HF itself was the main cause of death, even though infections, myocardial infarction, and pulmonary embolism represented a significant proportion of the cases. Regarding specific clinical scenarios, we found that in patients with sudden cardiac death, autopsy could identify a plausible cause of death in 43.7% of the patients. Furthermore, patients with preserved ejection fraction had a higher frequency of noncardiovascular causes of death compared to patients with reduced ejection fraction.

The high rate of diagnostic discrepancies (39.2%) involving the major diagnosis is comparable to published rates in other scenarios. In a study with 1800 autopsies performed between 1998 and 2008, discrepancies involving the major diagnosis were found in 43.4% of the patients in 1998; the rate of discrepancies had decreased to 27.1% in 2008.^[5]^ Another study sought to estimate the rate of discrepancies in 300 autopsies during 3 decades and found a reduction in the rate of discrepancies: 30% in 1973, 18% in 1982, and 14% in 1992.^[17]^ However, it should be acknowledged that these analyses were performed in less selected populations and are not specific to HF patients. In this regard, it is interesting that the presence of cardiovascular diseases was described as a risk factor for the occurrence of discrepancies.^[5]^ Moreover, in studies that included more severely ill patients, higher rates of discrepancies were reported, ranging between 18.5 and 39%.^[12,18]^ In accordance with our findings, a previous study performed at our institution found that 30% of patients with cardiovascular diseases were categorized as classes I and II.^[15]^ Taken together, these data point to the fact that diagnostic discrepancies are frequent in medical practice, especially among patients with cardiovascular diseases and HF.

Pulmonary embolism was the main cause of death in patients with discrepancies involving the major diagnosis. Clinical studies have suggested that up to 9% of the patients admitted with HF develop symptomatic pulmonary embolism during hospitalization. Data for a subclinical episode are more scarce, but typical estimates of the prevalence of pulmonary embolism in patients admitted due to HF range from 5% to 23%.^[19,20]^ Conversely, in large pulmonary embolism registries, HF was observed in 10.5% of patients and was found to be an independent mortality risk factor.^[21]^ An autopsy study of 111 patients with Chagas’ cardiomyopathy reported pulmonary embolism in 41 (36.9%) cases.^[22]^ These data point to the importance of pulmonary embolism in the acute HF setting, especially in high-risk populations, such as patients with Chagas’ cardiomyopathy and rheumatic disease.

We found that for each increase in 1 year of age, the chance of a major discrepancy increased 3%; conversely, the presence of diabetes mellitus was unexpectedly associated with a 64% reduction in the chance of major discrepancies. A previous study from our institution^[15]^ also found increasing age to be a risk factor for the occurrence of discrepancies. The same was found in a European study among women, but this finding was not confirmed among men.^[5]^ Death in wards (compared to intensive care units), death in community hospitals (compared to university hospitals), and the presence of cardiovascular disease are associated with an increased chance of discrepancies.^[5,15]^ The association between diabetes mellitus and diagnostic discrepancies has not been previously reported. Even though the present study did not explore mechanisms of this association, one can hypothesize that patients with diabetes mellitus could have undergone more thorough diagnostic investigation compared to patients without diabetes. Additionally, patients with diabetes mellitus mainly have cardiovascular causes of death, which may have favored the in vivo diagnosis of the processes related to death. In older patients, the presence of unspecific symptoms and the concurrence of multiple diseases may impose a diagnostic challenge; moreover, physicians may be less prone to perform extensive diagnostic investigation in older patients.

We also studied patients who experienced sudden death and found that in most cases (40.6%) there were signs of HF and a state of systemic low output, a finding compatible with the occurrence of fatal arrhythmias.^[23]^ In fact in 7 of these patients, an arrhythmic cause of death had been identified while the patients were alive. A substudy of the ATLAS trial^[24]^ analyzed 171 patients who experienced sudden death and were autopsied and found an acute coronary finding in 33% of the patients, a higher rate than that found in our study. This difference can be attributed to the heterogeneity between the studies regarding the prevalence of coronary artery disease in the populations considered. Additionally, it should be acknowledged that in our study the overall rate of sudden death was small, when compared to most cohorts and clinical trials that included patients with HF. Possible reasons for this finding are the inclusion of a population with more advanced HF, known to have lower frequencies of arrhythmic death and the inclusion of a significant proportion of patients with preserved ejection fraction.

When patients were categorized according to the status of ventricular function; patients with preserved ejection fraction had a higher proportion of female patients and were more likely to have arterial hypertension and rheumatic valvar disease as the etiology of HF. Additionally, we observed that patients with preserved ejection fraction tended to have a higher proportion of noncardiovascular causes of death, especially infections (25.6%), even though the difference was not statistically significant. Epidemiological studies indicate that cardiovascular conditions are responsible for 51% to 60% of deaths among patients with preserved ejection fraction.^[25]^

We believe that the present findings have important clinical implications. Firstly, they raise an epidemiological concern, given the large populational burden of HF. In United States^[7]^ in 2009, HF with any-mention of mortality was 274,601, and HF was the underlying cause in 56,410 of those deaths. Additionally, hospital discharges for HF from 2000 to 2010 were 1.008 million and 1.023 million, respectively, with an in-hospital mortality rate of 5.1%.^[26]^ The possibility that in more than 30% of deaths a diagnostic discrepancy may have been involved provides an impressive perspective on the paramount importance of this matter. Additionally, our findings point to the necessity of a revision of the decision-making process involving patients with HF, because such patients challenge the current presumption that the astounding advances in imaging techniques, endoscopies, and laboratory tests are almost infallible and warrant the development of new strategies to improve patient safety, quality of care, and health outcomes.^[27,28]^ In this sense, autopsy reports represent a unique opportunity to explore essential elements regarding patient safety,^[29]^ an issue that has gained momentum with the widespread attention toward diagnostic performance and care delivery.^[30]^ Autopsy analysis may particularly point to ways of preventing serious conditions eventually related to death, as well as indicate improvements in medical diagnostic rationale and frameworks. However, barriers to a more widespread use of autopsies in this scenario include lack of proper financial support, family reluctance in accepting the procedure, decreased medical interest as compared to more modern and less invasive diagnostic techniques, and medical hesitation in face of the possibility of professional errors.^[31]^

## 5 Conclusions

Our results indicate that a high proportion of the deaths can be attributed to diagnostic limitations and, thus, are potentially avoidable and that events not directly associated with the presence of myocardial dysfunction are responsible for a significant proportion of deaths in patients with HF who undergo autopsy. These findings warrant reconsiderations regarding the diagnostic workup and therapeutic interventions in patients with advanced HF.

### 5.1 Limitations

As an autopsy-based study, it should be acknowledged that a natural selection bias might have occurred, because autopsies tend to be more frequently performed in patients with a higher degree of diagnostic uncertainty. Furthermore, we could not retrieve clinical data for all patients who were autopsied. The inclusion of a high number of patients with Chagas disease, and of a low number of patients with ischemic heart disease as compared to cohorts^[32]^ and clinical trials^[33]^ in other HF populations are important aspects to be acknowledged.

### 5.2 Compliance with ethical standards

The study was reviewed and approved by institutional Ethics Committee.
